# Supplementary material for: Predicting infectivity: comparing four PCR‐based assays to detect culturable SARS‐CoV‐2 in clinical samples
Source: EMBO Mol Med. 2021 Dec 13;14(2):e15290. doi: 10.15252/emmm.202115290 (PMC8819313; doi:10.15252/emmm.202115290)
Supplement: Supplementary file 1 — Appendix [file EMMM-14-e15290-s002.pdf]

Appendix Table of Contents:

The appendix contains only one figure, Appendix Figure S1.

Fig S1

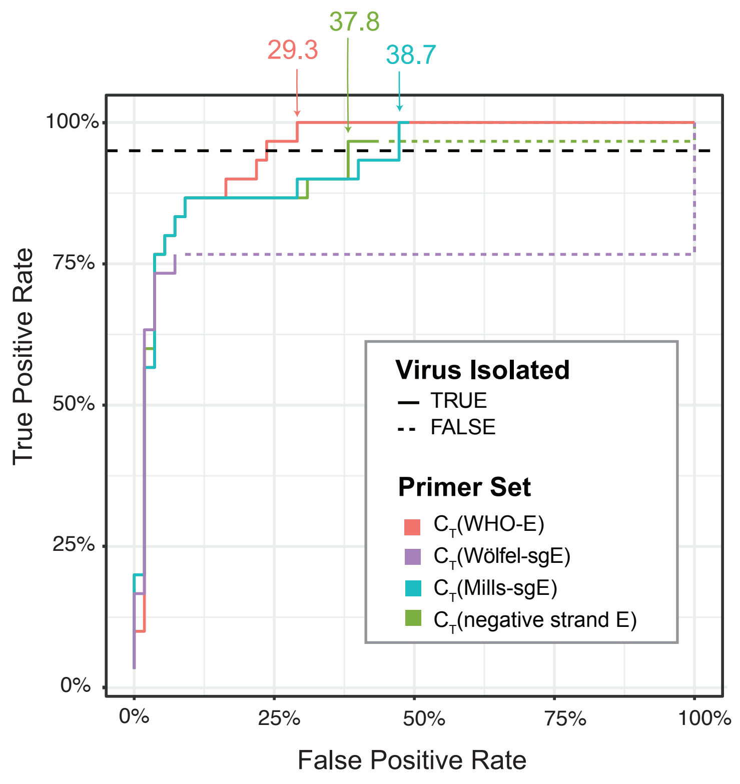

29.3
37.8
38.7
100%
75%
50%
25%
0%
True Positive Rate
0%
25%
50%
75%
100%
False Positive Rate
Virus Isolated
TRUE
FALSE
Primer Set
C_T(WHO-E)
C_T(Wölfel-sgE)
C_T(Mills-sgE)
C_T(negative strand E)

100%

Appendix Figure S1. Receiver operating characteristic (ROC) curve comparing diagnostic performance of WHO-E, Wölfel-sgE, Mills-sgE and negative-strand E primer-probe sets.

ROC curves showing the ability to determine infectivity (as assayed by viral culture in Vero E6-TMPRSS2 cells) using the C_T values of WHO-E, Wölfel-sgE, Mills-sgE, and negative-strand E for a subset of the samples from Fig 7 for which we had sufficient material to test these four primer sets. For each test, the true-positive rate (chance of correctly predicting samples to be culturable at a given or lower C_T) was plotted against the false-positive rate (chance of incorrectly predicting samples to be culturable at a given or lower C_T) as a step function line. The lines show performance of the tests as the (C_T) threshold for a positive result is increased; the lines are solid for detectable values and dashed when a primer set returned an undetectable value. Each line is labelled with the lowest C_T value that surpassed a 95% sensitivity threshold (the horizontal dashed line) for that marker.
