# Supplementary material for: Predicting infectivity: comparing four PCR‐based assays to detect culturable SARS‐CoV‐2 in clinical samples
Source: EMBO Mol Med. 2021 Dec 13;14(2):e15290. doi: 10.15252/emmm.202115290 (PMC8819313; doi:10.15252/emmm.202115290)
Supplement: Supplementary file 4 [file EMMM-14-e15290-s001.pdf]

EMBO Molecular Medicine

Predicting Infectivity:Comparing Four PCR Assays to Detect Culturable SARS-CoV-2 in Clinical Samples

Emily Bruce, Margaret Mills, Reigran Sampoleo, Garrett Perchetti, Meei-Li Huang, Hannah Despres, Madaline Schmidt, Pavitra Roychoudhury, David Shirley, Keith Jerome, Alexander Greninger, and Jason Botten
DOI: 10.15252/emmm.202115290

Corresponding authors: Emily Bruce (Emily.Bruce@med.uvm.edu) , Alexander Greninger (agrening@uw.edu), Jason Botten (jbotten@uvm.edu)

Review Timeline:

Transfer from Review Commons:	14th Oct 21
Editorial Decision:	10th Nov 21
Revision Received:	25th Nov 21
Accepted:	26th Nov 21

Editor: Zeljko Durdevic

Transaction Report:

This manuscript was transferred to EMBO Molecular Medicine following peer review at Review Commons.

(Note: With the exception of the correction of typographical or spelling errors that could be a source of ambiguity, letters and reports are not edited. Depending on transfer agreements, referee reports obtained elsewhere may or may not be included in this compilation. Referee reports are anonymous unless the Referee chooses to sign their reports.)

Review #1

1. How much time do you estimate the authors will need to complete the suggested revisions:

Estimated time to Complete Revisions (Required)

(Decision Recommendation)

Less than 1 month

2. Evidence, reproducibility and clarity:

Evidence, reproducibility and clarity (Required)

Summary:

Provide a short summary of the findings and key conclusions (including methodology and model system(s) where appropriate).
Authors developed a novel primer/probe set for detection of subgenomic (sgE) transcripts for SARS-CoV-2 with the aim to develop a system that may predict the presence of infectious virus in patient samples. After studying the specificity and sensitivity of their system, they compared it with already validated/published systems for diagnostic of SARS-CoV-2 infection. Interestingly, they also studied the effect of the conditions of isolation. They showed Vero E6 expressing TMPRSS2 (Vero E6-TMPRSS2) to be more sensitive to infection than Vero E6, allowing a higher number of isolation from patient samples. They also showed their system to be more sensitive than a previously published sgE system as well as than a negative-strand RNA assay but less sensitive than the WHO/Charité primer/probe set. Anyway, all samples containing infectious particles (successful virus isolation on Vero E6-TMPRSS2) were detected with their primer/probe system contrary to the other tested sgE assay. They showed the negative strand assay to be unlikely to detect virus genetic material in samples which nevertheless contain infectious particles.

Major comments:

-Are the key conclusions convincing?

I salute the intention of the authors to try to fix cut-off values for infectious patients but I would be more careful on the assertion of "using a total viral RNA Ct cut-off of >31 or specifically testing for sgRNA can serve as an effective rule-out test for viral infectivity". It is true that in this study, virus was not isolated from any of the samples below a Ct of 31 or negative in the developed sgE assay but all those assays are done on cell culture. We do not know how the transmission could occur for those samples from human to human. Being able to fix a cut-off in Ct value for a define PCR/RT-PCR system would be a great improvement for SARS-CoV-2 infected patient having to stay in quarantine. It is

even more important for Ebola positive patients in Africa who has to stay in quarantine in precarious conditions under tents, warm temperatures and without privacy for long period because they still positive by RT-PCR. Unfortunately, fix those values would need a very high number of experiments, including animal experiment.

-Should the authors qualify some of their claims as preliminary or speculative, or remove them altogether?
No

-Would additional experiments be essential to support the claims of the paper? Request additional experiments only where necessary for the paper as it is, and do not ask authors to open new lines of experimentation.
No

-Are the suggested experiments realistic in terms of time and resources? It would help if you could add an estimated cost and time investment for substantial experiments.
Yes.

-Are the data and the methods presented in such a way that they can be reproduced?

-Kinetic of SARS-CoV-2 (figure 2):
The method is not detailed in the Methods part and is not clear in the figure legend. When supernatant are collected, is it all the supernatant that is remove? An aliquot? If aliquot, do you replace with new medium?
-Stability of infectious SARS-CoV-2:
I am very surprise by your results on stability of cultured virus, knowing we observed a decreased of SARS-CoV-2 titer in our lab after freezing/thawing steps. Do you freeze cell supernatant directly or do you prepare your samples another way? Please state it in the Methods part

-Are the experiments adequately replicated and statistical analysis adequate?
Yes

Minor comments:

- Specific experimental issues that are easily addressable.

Figure 2C and D: Instead of Ct values in cells, it would be more relevant to normalize these results with an endogenous gene and present results as fold change to mock-infected cells. Because you affirm that the level of RNA decline than stay stable over the time but you also note there is CPE. If you have less cells but same level of viral RNA, it means you have an increase in the RNA level in alive cells.
It would have been interesting to have the results of isolation at different time-point of treatment for patient samples (figure 3A and B) to see if the virus is stable in samples

-Are prior studies referenced appropriately? Yes

-Are the text and figures clear and accurate?
Yes.

Line 140: "this delay in virus and RNA production". You do not talk about RNA yet...

Line 156 to 163: sgE RNA detected in cell free supernatant. Can't it come from lysed cells?

Line 167: "...virus in cell culture time course experiment in TMPRRS2 expressing cells (fig.2)"

Ligne 258: Fig 6A and B

-Do you have suggestions that would help the authors improve the presentation of their data and conclusions? No

3. Significance:

Significance (Required)

-Describe the nature and significance of the advance (e.g. conceptual, technical, clinical) for the field.

This new primer/probe system will participate to the accurate diagnostic of SARS-CoV-2. The comparison with the existing methods is relevant to highlight the strengths and weaknesses of each system. Comparison of isolation of SARS-CoV-2 on commonly used Vero E6 with Vero E6-TMPRSS2 will lead to a great improvement of the isolation method for SARS-CoV-2.

-Place the work in the context of the existing literature (provide references, where appropriate).
Properly done in the introduction of the paper.

-State what audience might be interested in and influenced by the reported findings.
Diagnostic laboratories

-Define your field of expertise with a few keywords to help the authors contextualize your point of view. Indicate if there are any parts of the paper that you do not have sufficient expertise to evaluate.
Virology, Molecular Biology, cell biology
Not enough expertise to evaluate ROC data/analysis

Review #2

1. How much time do you estimate the authors will need to complete the suggested revisions:

Estimated time to Complete Revisions (Required)

(Decision Recommendation)

Between 1 and 3 months

2. Evidence, reproducibility and clarity:

Evidence, reproducibility and clarity (Required)

Summary:

Bruce et al present a new RT-PCR assay with primer sets that specifically detect sgE RNA from SARS-CoV2 samples. The authors compare this assay to other diagnostic assays in an effort to identify assays capable of correlating RNA detection with culturable virus (i.e. infectious virus). While this new assay identified 100% of culturable isolates, only 56% of isolates testing positive actually had culturable virus. Compared with other assays, the WHO total E RNA assay had better parameters when used at a cutoff Ct value of 31 (PPV of 61%). Overall, this manuscript provides a novel primer probe set for RT-PCR diagnostic assay and conducted comparisons with other assays on the same clinical samples. There are some areas that the authors should address prior to publication.

Major comments:

-The authors repeatedly tout VeroE6 TMRSS2 cells as supporting higher viral infection. Therefore, the authors should address why one clinical isolate (E16) was culturable in VeroE6 but not VeroE6 TMRSS2. Was this experiment repeated multiple times? What are the reasons for this discrepancy?

-The authors' argument at lines 166-169 is not supported by the data in Fig. 2. The levels of viral RNA between VeroE6 and VeroE6 TMRSS2 appear to show similar trends in the supernatant across the time course but the infectious viral levels are dramatically different. This discordance between FFU levels and RNA levels cannot be explained by instability of viral particles alone. Have the authors looked into differences in viral particles produced from these two cell lines? The authors should collect virus particles from these two cell lines and conduct the stability experiment in Fig 2D to directly test the hypothesis that indeed the drop seen in FFU in VeroE6 TMRSS2 is due to instability.

-The evidence for the packaging of sgE RNA into virions is weak. GAPDH detection by PCR is not a proof that the concentration process did not pellet RNA nonspecifically. First, the authors should provide ample information about viral isolation process at line 379 including rotor, centrifuge and speed utilized. In addition, ribosomes typically stay intact following viral lysis (and can be found in supernatant after release from dead cells). Actively translating ribosomes can contain sgE RNA as well. The authors should consider detecting ribosomal RNAs in their samples to rule out the possibility of contaminating ribosomes. In addition, the authors should strongly consider repeating the experiment with high EDTA concentration to break up ribosomes and only pellet

virions.

Minor comments:

-At line 197, the authors refer to "viruses" with lower levels of SARS-CoV2 RNA. This is incorrect and should be changed to "isolates" as the SARS-CoV2 virus particle does not package variable amount of genomic RNA.

-The authors statement on lines 210-212 does not seem to be supported clearly by Fig. 5. The authors should consider including trendlines as well as other analyses that help show the correlation between viral RNA vs FFU. In addition, the authors should label the Y-axis clearly for Fig. 5.

-The authors should expand on the methodology for creating ROC curves at line 467.

3. Significance:

Significance (Required)

This study is significant because it assesses the utility of several clinical assays for the measurement of viral RNA and correlating it with culturable virus. This is important in the field because it helps to identify methods whereby infectivity can be predicted from a simple diagnostic test. This is important to know as a virologist working in the SARS-CoV2 field. It is also important from a public health perspective to better define quarantine requirements for persons testing positive. While the study provided a new primer probe set, it appears that the already available WHO total E RNA assay is superior in predicting infectivity and this study provides further evidence to support this notion.

Full Revision

Manuscript number: #RC-2021-00951

Corresponding author(s): Emily Bruce, Alexander Greninger, and Jason Botten

[Please use this template only if the submitted manuscript should be considered by the affiliate journal as a full revision in response to the points raised by the reviewers.

If you wish to submit a preliminary revision with a revision plan, please use our "Revision Plan" template. ***It is important to use the appropriate template to clearly inform the editors of your intentions.****]*

1. General Statements [optional]

This section is optional. Insert here any general statements you wish to make about the goal of the study or about the reviews.

We would like to thank the Editor(s) and Reviewers for their time evaluating our submission. We appreciate their insightful suggestions. Both reviewers acknowledged the timely significance of the study and confirmed that we carried out experiments with the appropriate methodologies and rigor. By directly addressing their critiques, we believe the manuscript has been strengthened, with conclusions that are strongly supported by the data.

2. Point-by-point description of the revisions

This section is mandatory. Please insert a point-by-point reply describing the revisions that were already carried out and included in the transferred manuscript.

Reviewer #1 (Evidence, reproducibility and clarity (Required)):

Summary:

Provide a short summary of the findings and key conclusions (including methodology and model system(s) where appropriate).
Authors developed a novel primer/probe set for detection of subgenomic (sgE) transcripts for SARS-CoV-2 with the aim to develop a system that may predict the presence of infectious virus in patient samples. After studying the specificity and sensitivity of their system, they compared it with already validated/published systems for diagnostic of SARS-CoV-2 infection. Interestingly, they also studied the effect of the conditions of isolation. They showed Vero E6 expressing TMPRSS2 (Vero E6-TMPRSS2) to be more sensitive to infection than Vero E6, allowing a higher number of isolation from patient samples. They also showed their system to be more

sensitive than a previously published sgE system as well as than a negative-strand RNA assay but less sensitive than the WHO/Charité primer/probe set. Anyway, all samples containing infectious particles (successful virus isolation on Vero E6-TMPRSS2) were detected with their primer/probe system contrary to the other tested sgE assay. They showed the negative strand assay to be unlikely to detect virus genetic material in samples which nevertheless contain infectious particles.

Major comments:

-Are the key conclusions convincing?

I salute the intention of the authors to try to fix cut-off values for infectious patients but I would be more careful on the assertion of "using a total viral RNA Ct cut-off of >31 or specifically testing for sgRNA can serve as an effective rule-out test for viral infectivity". It is true that in this study, virus was not isolated from any of the samples below a Ct of 31 or negative in the developed sgE assay but all those assays are done on cell culture. We do not know how the transmission could occur for those samples from human to human. Being able to fix a cut-off in Ct value for a define PCR/RT-PCR system would be a great improvement for SARS-CoV-2 infected patient having to stay in quarantine. It is even more important for Ebola positive patients in Africa who has to stay in quarantine in precarious conditions under tents, warm temperatures and without privacy for long period because they still positive by RT-PCR. Unfortunately, fix those values would need a very high number of experiments, including animal experiment.

We appreciate the reviewer's acknowledgment of the significance of this issue. We agree that *in vivo* animal experiments to more precisely determine the lowest infectious or transmissible dose would be valuable. But such experiments are outside the scope of the current study. To acknowledge the reviewer's important point regarding the unavoidable limitations of cell culture systems, we have modified the abstract (line 51) to say “an effective rule out test for the presence of culturable virus,” a conclusion that is fully supported by our data.

-Should the authors qualify some of their claims as preliminary or speculative, or remove them altogether?
No

-Would additional experiments be essential to support the claims of the paper? Request additional experiments only where necessary for the paper as it is, and do not ask authors to open new lines of experimentation.
No

-Are the suggested experiments realistic in terms of time and resources? It would help if you could add an estimated cost and time investment for substantial experiments.
Yes.

-Are the data and the methods presented in such a way that they can be reproduced?

-Kinetic of SARS-CoV-2 (figure 2):
The method is not detailed in the Methods part and is not clear in the figure legend. When supernatant are collected, is it all the supernatant that is remove? An aliquot? If aliquot, do you replace with new medium?
We apologize for this omission and have included the requested details in the methods. We seed a separate well for each time point and collected the entire supernatant for a given time point, rather than replacing media. We added the following text to the methods section (lines 402-412): *"Viral growth kinetics were measured in Vero E6 or Vero E6 TMPRSS2 cells at an MOI of 0.001. Separate wells were seeded for each time point, and growth curves were conducted in technical duplicates for each biological experiment. Supernatants and cell lysates were collected twice daily 1 & 2 dpi, and again on 3, 4, 7 and 8 dpi (Vero E6 TMPRSS2 cells were harvested for the final time at day 7 due to faster growth kinetics in this cell type). For each time point, the supernatant was removed and clarified to remove cellular debris, before being split into separate aliquots for RNA extraction (mixed 1:1 with AVE lysis buffer) and viral titration (by focus assay). Dead cells/debris that was pelleted after clarifying supernatants was combined with cells scraped from each well into PBS and spun again to obtain a pellet of all cell material from each timepoint. This pellet was then lysed in AVE viral lysis buffer for RNA extraction."*

-Stability of infectious SARS-CoV-2:
I am very surprise by your results on stability of cultured virus, knowing we observed a decreased of SARS-CoV-2 titer in our lab after freezing/thawing steps. Do you freeze cell supernatant directly or do you prepare your samples another way? Please state it in the Methods part

We measured the stability after freeze/thaw for our normal high concentration viral stocks. Our viral stocks are grown in DMEM with 10% FBS, 1% HEPES, 1% pen/strep, and clarified before use. It is possible that lab-lab variation in the media components or HEPES concentration used to prepare viral stocks explains the differences seen in our work vs the reviewer's lab. We have added the following additional detail to the methods section (lines 415-418) of the manuscript to clarify how these experiments were performed: "*High concentration viral stocks (prepared as above in DMEM, 10% FBS, 1% HEPES, 1% pen/strep) were used to measure viral stability over time and after multiple freeze-thaw cycles. Stocks were stored at the indicated temperatures in the dark and aliquots were removed at the indicated days or after each freeze-thaw cycle for measuring infectious virus by focus assay."*

-Are the experiments adequately replicated and statistical analysis adequate?
Yes

Minor comments:

- Specific experimental issues that are easily addressable.

Figure 2C and D: Instead of Ct values in cells, it would be more relevant to normalize these results with an endogenous gene and present results as fold change to mock-infected cells. Because you affirm that the level of RNA decline than stay stable over the time but you also

note there is CPE. If you have less cells but same level of viral RNA, it means you have an increase in the RNA level in alive cells.

We have measured the GAPDH level in these cells over time, and that data is included as gray lines in Fig 2 C&D (pasted below). As we are combining the cell pellet from clarified supernatants with the cells that remain adherent to the dish for each harvested timepoint we expect to be harvesting the majority of cells/cell debris for each time point. The levels of GAPDH remain broadly similar over the viral growth curve, with no drop in RNA levels.

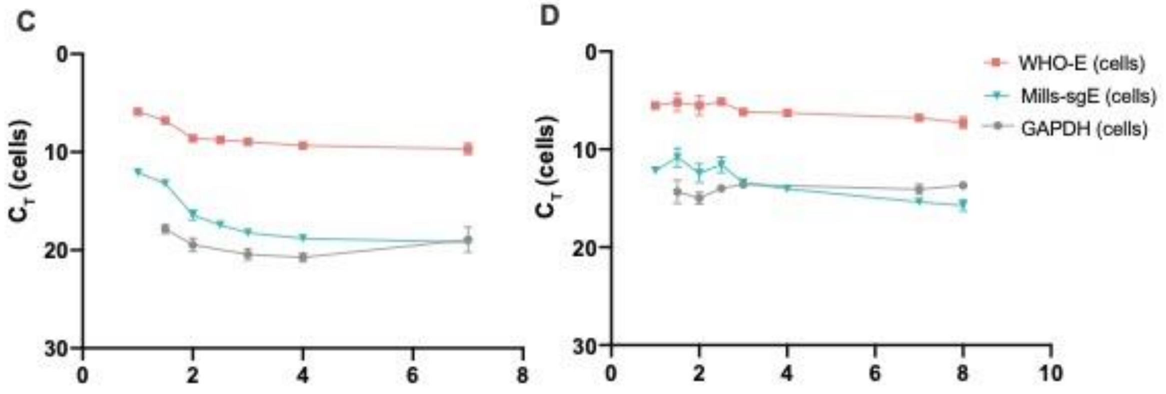

It would have been interesting to have the results of isolation at different time-point of treatment for patient samples (figure 3A and B) to see if the virus is stable in samples

We have access to only limited volume (several hundred µl) of residual patient sample which would make it technically challenging to compare multiple days of storage conditions/ temperatures. Unfortunately, we do not have any remaining sample volume for the specimens used in this study, and so we are unable to perform additional isolations at other times/temperatures. While we agree this would be an interesting line of future inquiry, we feel it is outside the scope of the current study.

-Are prior studies referenced appropriately? Yes

-Are the text and figures clear and accurate?
Yes.

Line 140: "this delay in virus and RNA production". You do not talk about RNA yet...

We have removed “and RNA” from this sentence and replaced with “infectious virus production”.

Line 156 to 163: sgE RNA detected in cell free supernatant. Can't it come from lysed cells?

We have replaced “cell-free” with “clarified”.

Line 167: "...virus in cell culture time course experiment in TMPRRS2 expressing cells (fig.2)"

We have modified this text to read according to the Reviewer's suggestion.

Ligne 258: Fig 6A and B

We have added the missing reference to Fig 6B as requested.

-Do you have suggestions that would help the authors improve the presentation of their data and conclusions? No

Reviewer #1 (Significance (Required)):

-Describe the nature and significance of the advance (e.g. conceptual, technical, clinical) for the field.

This new primer/probe system will participate to the accurate diagnostic of SARS-CoV-2. The comparison with the existing methods is relevant to highlight the strengths and weaknesses of each system. Comparison of isolation of SARS-CoV-2 on commonly used Vero E6 with Vero E6-TMPRSS2 will lead to a great improvement of the isolation method for SARS-CoV-2.

We appreciate the Reviewer's assessment of the significance of our study and the improvement in our isolation method compared to the existing standard of using Vero E6 cells.

-Place the work in the context of the existing literature (provide references, where appropriate). Properly done in the introduction of the paper.

-State what audience might be interested in and influenced by the reported findings.
Diagnostic laboratories

-Define your field of expertise with a few keywords to help the authors contextualize your point of view. Indicate if there are any parts of the paper that you do not have sufficient expertise to evaluate.
Virology, Molecular Biology, cell biology
Not enough expertise to evaluate ROC data/analysis

Reviewer #2 (Evidence, reproducibility and clarity (Required)):

Summary:

Bruce et al present a new RT-PCR assay with primer sets that specifically detect sgE RNA from SARS-CoV2 samples. The authors compare this assay to other diagnostic assays in an effort to identify assays capable of correlating RNA detection with culturable virus (i.e. infectious virus).

While this new assay identified 100% of culturable isolates, only 56% of isolates testing positive actually had culturable virus. Compared with other assays, the WHO total E RNA assay had better parameters when used at a cutoff Ct value of 31 (PPV of 61%). Overall, this manuscript provides a novel primer probe set for RT-PCR diagnostic assay and conducted comparisons with other assays on the same clinical samples. There are some areas that the authors should address prior to publication.

Major comments:

-The authors repeatedly tout VeroE6 TMRSS2 cells as supporting higher viral infection. Therefore, the authors should address why one clinical isolate (E16) was culturable in VeroE6 but not VeroE6 TMRSS2. Was this experiment repeated multiple times? What are the reasons for this discrepancy?

We did not have sufficient residual sample volume to repeat isolation attempts of any clinical specimen, so we are limited to a single data point for each cell line. It is possible that this sample had levels of infectious virus at the limit of detection, and stochastic probability meant infectious virus was only present in the aliquot used to infect the Vero E6 (rather than Vero E6-TMPRSS2) cells. It is also possible that viral adaptation/evolution occurred in the VeroE6 well that allowed this virus to successfully grow, but we do not have sequencing data or remaining nucleic acids to test this theory.

-The authors' argument at lines 166-169 is not supported by the data in Fig. 2. The levels of viral RNA between VeroE6 and VeroE6 TMRSS2 appear to show similar trends in the supernatant across the time course but the infectious viral levels are dramatically different. This discordance between FFU levels and RNA levels cannot be explained by instability of viral particles alone. Have the authors looked into differences in viral particles produced from these two cell lines? The authors should collect virus particles from these two cell lines and conduct the stability experiment in Fig 2D to directly test the hypothesis that indeed the drop seen in FFU in VeroE6 TMRSS2 is due to instability.

We apologize for the confusion. We did not intend to make claims about differences in particle stability as a result of the cell line used for viral production, but rather to highlight a general observation that RNA was more stable than infectious virus. This is more obvious in the TMRPSS2 cell line, as replication is faster and more synchronized than in Vero E6 cells (the TMRPSS2 cells are largely dead by day 4, whereas infection progresses more slowly in Vero E6 cells so that new virions continue to be produced during the measured time period). We have added clarifying text at line 167-169, "*We observed that SARS-CoV-2 RNA species persist for much longer than infectious virus in cell culture time course experiments, a feature that was most obvious in Vero E6 TMRPSS-2 cells due to their viral kinetics but is likely not cell specific (Fig 2).*"

-The evidence for the packaging of sgE RNA into virions is weak. GAPDH detection by PCR is

not a proof that the concentration process did not pellet RNA nonspecifically. First, the authors should provide ample information about viral isolation process at line 379 including rotor, centrifuge and speed utilized. In addition, ribosomes typically stay intact following viral lysis (and can be found in supernatant after release from dead cells). Actively translating ribosomes can contain sgE RNA as well. The authors should consider detecting ribosomal RNAs in their samples to rule out the possibility of contaminating ribosomes. In addition, the authors should strongly consider repeating the experiment with high EDTA concentration to break up ribosomes and only pellet virions.

We have added additional experimental details (rotor, centrifuge and speed) describing how the viral concentration step was performed (line 389-394), "*Viral RNA (courtesy of David Bauer, The Francis Crick Institute, UK) from concentrated SARS-CoV-2 (England02 strain, B lineage 'Wuhan-like') was obtained by clarifying viral supernatants (2 x 4000 rpm for 30 mins at 4°C in a Beckman Allegra X-30R centrifuge with a SX4400 rotor), overlaying clarified media onto a 30% sucrose/PBS cushion (1/4th tube volume) and concentrating by ultracentrifugation in a Beckman ultra XPN-90 centrifuge with SW32TI rotor for 90 min at 25,500 rpm at 4°C. Pellets were then resuspended in buffer and extracted with TRIzol LS.*" We thank the reviewer for their suggestion of including an additional control, and we have added an 18S primer-probe set (see new Figure 8). This data, while not as pronounced as the GAPDH control, suggests that the ultracentrifugation step has removed significant amounts of 18S RNA (though the clarified supernatants retain similar amounts of 18S RNA as the cells, suggesting that clarification alone is not sufficient to remove contaminating ribosomes). While we agree that repeating the ultracentrifuge concentration with high concentrations of EDTA is an interesting line of inquiry we feel it is outside the scope of this manuscript (and we face additional technical restrictions to pursue this as we currently lack access to an ultracentrifuge at BSL-3). We have updated the discussion to include the possibility of residual ribosome-protected fragments of sgE as a potential alternative interpretation (line 350-352).

Minor comments:

-At line 197, the authors refer to "viruses" with lower levels of SARS-CoV2 RNA. This is incorrect and should be changed to "isolates" as the SARS-CoV2 virus particle does not package variable amount of genomic RNA.

We have changed this to "clinical specimens" for clarity.

-The authors statement on lines 210-212 does not seem to be supported clearly by Fig. 5. The authors should consider including trendlines as well as other analyses that help show the correlation between viral RNA vs FFU. In addition, the authors should label the Y-axis clearly for Fig. 5.

We have added clarifying labels to both the X and Y axes. Due to the limited sample volume we were unable to directly measure the infectious titers from the clinical samples used in this study,

and thus the FFU/mL represents the titer post-isolation while the C_T represents the amount of RNA pre-isolation. Nonetheless, we do see broad trends (ie, the colored dots are generally arranged in rainbow order from left to right, though we agree there is variation within this trend). We have also modified the text at lines 212-217 to reflect the reviewer's concern- "*Greater initial viral RNA levels was broadly associated with faster viral growth in both cell lines (seen in the progression of colors from left to right), however we saw significant variation within these trends. Our data suggests that when standard SARS-CoV-2 RNA RT-PCR values are the only available data for patient or population-level viral loads, they are useful in gauging the presence of infectious virus in patient NP samples (Fig 5).*"

-The authors should expand on the methodology for creating ROC curves at line 467.

We have included the following text in the methods section for ROC curve analysis:

"ROC curves were generated using R [43]. For each potential scoring marker (CT_e, CT_sge1, CT_sge2, neg_e,) samples were ordered by that marker, followed by culturable status. The false-positive rate was calculated as the cumulative count of culturable samples (after ordering by marker intensity) divided by the total count of culturable samples; the true positive rate was calculated as the cumulative count of non-culturable samples (after ordering) divided by the total count of non-culturable samples. The false positive rate was plotted on the X axis of the ROC curves and the true positive rate on the Y axis."

Reviewer #2 (Significance (Required)):

This study is significant because it assesses the utility of several clinical assays for the measurement of viral RNA and correlating it with culturable virus. This is important in the field because it helps to identify methods whereby infectivity can be predicted from a simple diagnostic test. This is important to know as a virologist working in the SARS-CoV2 field. It is also important from a public health perspective to better define quarantine requirements for persons testing positive. While the study provided a new primer probe set, it appears that the already available WHO total E RNA assay is superior in predicting infectivity and this study provides further evidence to support this notion.

We appreciate the Reviewer's assessment that this study is significant and provides information of high interest to SARS-CoV-2 virologists that also has important public health implications.

10th Nov 2021

Dear Dr. Bruce,

Thank you for the submission of your revised manuscript to EMBO Molecular Medicine. I am pleased to inform you that we will be able to accept your manuscript pending the following final amendments:

1) In the main manuscript file, please do the following:
- Correct/answer the track changes suggested by our data editors by working from the attached document.
- Add up to 5 keywords.
- Make sure that all special characters display well.
- Add author contributions.
- In M&M, a statistical paragraph that should reflect all information that you have filled in the Authors Checklist, especially regarding randomization, blinding, replication.
- In M&M, please specify the biosafety level for the experiments with SARS-CoV-2 by adding and amending the following sentence: All experiments with SARS-CoV-2 were performed in a ... level laboratory and with approval from...
- In M&M, include a statement from the the Authors Checklist that "The use of deindentified samples in this study was approved by the University of Washington Institutional Review Board under a consent waiver, and were determined to be exempt."
- Place "Acknowledgments", "Data availability" and "Conflict of interest" at the end of M&M section.
- In Data availability section please add URL that leads directly to the R code.
- Correct the reference citation. In the reference list, citations should be listed in alphabetical order. Where there are more than 10 authors on a paper, 10 will be listed, followed by "et al.". Please check "Author Guidelines" for more information. https://www.embopress.org/page/journal/17574684/authorguide#referencesformat
2) Funding: Department of Laboratory Medicine and Pathology, University of Washington is entered in our submission system, but this information is missing in the Acknowledgements. Please make sure that information about all sources of funding are complete in both our submission system and in the manuscript.
3) Supplemental data: In the manuscript text there are callouts for a Suppl. Figure 1 and Suppl. Tables 1 and 2, but the files are missing. Either compilet these data in a .pdf file with ToC and legends, rename suppl. figures and tables to "Appendix Figure S1" and "Appendix Tables S1 and S2", and update callouts in the text. If the tables are large, name them "Dataset EV1" and EV2 and upload as .xlsx dataset files. In that case they need their descriptions added in a separate tab of the file.
4) The Paper Explained: Please provide "The Paper Explained" and add it to the main manuscript text. Please check "Author Guidelines" for more information. https://www.embopress.org/page/journal/17574684/authorguide#researcharticleguide
5) Synopsis: Every published paper now includes a 'Synopsis' to further enhance discoverability. Synopses are displayed on the journal webpage and are freely accessible to all readers. They include separate synopsis image and synopsis text.
- Synopsis image: Please provide a striking image or visual abstract as a high-resolution jpeg file 550 px-wide x (250-400)-px high to illustrate your article.
- Synopsis text: Please provide a short stand first (maximum of 300 characters, including space) as well as 2-5 one sentence bullet points that summarise the paper as a .doc file. Please write the bullet points to summarise the key NEW findings. They should be designed to be complementary to the abstract - i.e. not repeat the same text. We encourage inclusion of key acronyms and quantitative information (maximum of 30 words / bullet point). Please use the passive voice.
- Please check your synopsis text and image before submission and submit their final versions with your revised manuscript. Please be aware that in the proof stage minor corrections only are allowed (e.g., typos).
6) For more information: There is space at the end of each article to list relevant web links for further consultation by our readers. Could you identify some relevant ones and provide such information as well? Some examples are patient associations, relevant databases, OMIM/proteins/genes links, author's websites, etc...
7) Source data: We encourage you to include the source data for figure panels that show essential data. Numerical data should be provided as individual .xls or .csv files (including a tab describing the data). For blots or microscopy, uncropped images should be submitted (using a zip archive if multiple images need to be supplied for one panel). Please check "Author Guidelines" for more information. https://www.embopress.org/page/journal/17574684/authorguide#sourcedata
8) Press release: Please inform us as soon as possible and latest at the time of submission of the revised manuscript if you plan a press release for your article so that our publisher could coordinate publication accordingly.
9) Please be aware that we use a unique publishing workflow for COVID-19 papers: a non-typeset PDF of the accepted manuscript is published as "Just Accepted" on our website. With respect to a possible press release, we have the option to not post the "Just Accepted" version if you prefer to wait with the press release for the typeset version. Please let us know whether you agree to publication of a "Just accepted" version or you prefer to wait for the typeset version.
10) As part of the EMBO Publications transparent editorial process initiative (see our Editorial at http://embomolmed.embopress.org/content/2/9/329), EMBO Molecular Medicine will publish online a Review Process File (RPF) to accompany accepted manuscripts. This file will be published in conjunction with your paper and will include the anonymous referee reports, your point-by-point response and all pertinent correspondence relating to the manuscript. Let us know whether you agree with the publication of the RPF and as here, if you want to remove or not any figures from it prior to publication. Please note that the Authors checklist will be published at the end of the RPF.

11) Please provide a point-by-point letter INCLUDING my comments as well as the reviewer's reports and your detailed responses (as Word file).

You can submit your revised files by logging onto our online manuscript tracking system or simply follow this link:

Link Not Available

Please do not share this URL as it will give anyone who clicks it access to your account.

I look forward to reading a new revised version of your manuscript as soon as possible.

Yours sincerely,

Zeljko Durdevic

***** Reviewer's comments *****

Referee #1 (Remarks for Author):

The authors adequately addressed all comments raised at the last review.

Referee #2 (Comments on Novelty/Model System for Author):

This new primer/probe system will participate to the accurate diagnostic of SARS-CoV-2. The comparison with the existing methods is relevant to highlight the strengths and weaknesses of each system. Comparison of isolation of SARS-CoV-2 on commonly used Vero E6 with Vero E6-TMPRSS2 will lead to a great improvement of the isolation method for SARS-CoV-2.

Referee #2 (Remarks for Author):

All the points highlighted during my pre-submission review (review commons) were properly revised/discussed. The same for the second reviewer comments. I don't have more comments for this manuscript.

***
Rev_Com_number: RC-2021-00951
New_manu_number: EMM-2021-15290
Corr_author: Bruce
Title: Predicting Infectivity: Comparing Four PCR-based Assays to Detect Culturable SARS-CoV-2 in Clinical Samples

***** Reviewer's comments *****

Referee #1 (Remarks for Author):

The authors adequately addressed all comments raised at the last review.
We thank Reviewer #1 for their time and suggestions to improve this manuscript.

Referee #2 (Comments on Novelty/Model System for Author):

This new primer/probe system will participate to the accurate diagnostic of SARS-CoV-2. The comparison with the existing methods is relevant to highlight the strengths and weaknesses of each system. Comparison of isolation of SARS-CoV-2 on commonly used Vero E6 with Vero E6-TMPRSS2 will lead to a great improvement of the isolation method for SARS-CoV-2.

Referee #2 (Remarks for Author):

All the points highlighted during my pre-submission review (review commons) were properly revised/discussed. The same for the second reviewer comments. I don't have more comments for this manuscript.
We thank Reviewer #2 for their time and suggestions to improve this manuscript, and are pleased that they feel this data will lead to improvements in isolation methods and diagnostics for SARS-CoV-2.

26th Nov 2021

Dear Dr. Bruce,

We are pleased to inform you that your manuscript is accepted for publication and is now being sent to our publisher to be included in the next available issue of EMBO Molecular Medicine.

EMBO PRESS

YOU MUST COMPLETE ALL CELLS WITH A PINK BACKGROUND

PLEASE NOTE THAT THIS CHECKLIST WILL BE PUBLISHED ALONGSIDE YOUR PAPER

Corresponding Author Name: Emily Bruce, Alexander Greninger, Jason Botten
Journal Submitted to: EMBO Molecular Medicine
Manuscript Number: EMM-2021-15290

Reporting Checklist For Life Sciences Articles (Rev. June 2017)

This checklist is used to ensure good reporting standards and to improve the reproducibility of published results. These guidelines are consistent with the Principles and Guidelines for Reporting Preclinical Research issued by the NIH in 2014. Please follow the journal's authorship guidelines in preparing your manuscript.

A- Figures
1. Data
The data shown in figures should satisfy the following conditions:

- the data were obtained and processed according to the field's best practice and are presented to reflect the results of the experiments in an accurate and unbiased manner.
- figure panels include only data points, measurements or observations that can be compared to each other in a scientifically meaningful way.
- graphs include clearly labeled error bars for independent experiments and sample sizes. Unless justified, error bars should not be shown for technical replicates.
- if n< 5, the individual data points from each experiment should be plotted and any statistical test employed should be justified
- Source Data should be included to report the data underlying graphs. Please follow the guidelines set out in the author ship guidelines on Data Presentation.

2. Captions
Each figure caption should contain the following information, for each panel where they are relevant:

- a specification of the experimental system investigated (eg cell line, species name).
- the assay(s) and method(s) used to carry out the reported observations and measurements
- an explicit mention of the biological and chemical entity(ies) that are being measured.
- an explicit mention of the biological and chemical entity(ies) that are altered/varied/perturbed in a controlled manner.
- the exact sample size (n) for each experimental group/condition, given as a number, not a range;
- a description of the sample collection allowing the reader to understand whether the samples represent technical or biological replicates (including how many animals, litters, cultures, etc.).
- a statement of how many times the experiment shown was independently replicated in the laboratory.
- definitions of statistical methods and measures:
  - common tests, such as t-test (please specify whether paired vs. unpaired), simple $\chi2$ tests, Wilcoxon and Mann-Whitney tests, can be unambiguously identified by name only, but more complex techniques should be described in the methods section;
  - are tests one-sided or two-sided?
  - are there adjustments for multiple comparisons?
  - exact statistical test results, e.g., P values = x but not P values < x;
  - definition of 'center values' as median or average;
  - definition of error bars as s.d. or s.e.m.

Any descriptions too long for the figure legend should be included in the methods section and/or with the source data.

In the pink boxes below, please ensure that the answers to the following questions are reported in the manuscript itself. Every question should be answered. If the question is not relevant to your research, please write NA (non applicable). We encourage you to include a specific subsection in the methods section for statistics, reagents, animal models and human subjects.

USEFUL LINKS FOR COMPLETING THIS FORM

http://www.antibodypedia.com
http://1degreebio.org
http://www.equator-network.org/reporting-guidelines/improving-bioscience-research-repor
http://grants.nih.gov/grants/olaw/olaw.htm
http://www.mrc.ac.uk/Ourresearch/Ethicsresearchguidance/Useofanimals/index.htm
http://ClinicalTrials.gov
http://www.consort-statement.org
http://www.consort-statement.org/checklists/view/32-consort/66-title
http://www.equator-network.org/reporting-guidelines/reporting-recommendations-for-tumo
http://datadryad.org
http://figshare.com
http://www.ncbi.nlm.nih.gov/gap
http://www.ebi.ac.uk/ega
http://biomodels.net/
http://biomodels.net/miriam/
http://jjj.biochem.sun.ac.za
http://oba.od.nih.gov/biosecurity/biosecurity_documents.html
http://www.selectagents.gov/

B- Statistics and general methods

Please fill out these boxes (Do not worry if you cannot see all your text once you press return)

1.a. How was the sample size chosen to ensure adequate power to detect a pre-specified effect size?	Sample size was based on previous literature and what was feasible to obtain during a period of high positivity rates and limited testing capability in the Seattle area.
1.b. For animal studies, include a statement about sample size estimate even if no statistical methods were used.	There are no animal studies in this manuscript.
2. Describe inclusion/exclusion criteria if samples or animals were excluded from the analysis. Were the criteria pre-established?	The only samples excluded from analysis are listed in the supplemental dataset, and were excluded because CPE was likely the result of contamination from neighboring wells during isolation (based on the kinetics, plate placement and initial RNA levels of samples during the course of isolation)
3. Were any steps taken to minimize the effects of subjective bias when allocating animals/samples to treatment (e.g. randomization procedure)? If yes, please describe.	No samples were allocated to specific groups.
For animal studies, include a statement about randomization even if no randomization was used.	NA
4.a. Were any steps taken to minimize the effects of subjective bias during group allocation or/and when assessing results (e.g. blinding of the investigator)? If yes please describe.	RNA level (cycle threshold) of clinical specimens was blinded from investigator performing BSL3 titer work.
4.b. For animal studies, include a statement about blinding even if no blinding was done	NA
5. For every figure, are statistical tests justified as appropriate?	The Fisher Exact test used is appropriate for a 2x2 contingency table and provides exact p values.
Do the data meet the assumptions of the tests (e.g., normal distribution)? Describe any methods used to assess it.	The Fisher Exact test assumes that the binary data are independent, and does not make any assumptions about normality. The data were drawn from independent biological samples.
Is there an estimate of variation within each group of data?	Graphs show SEM; estimation of within-group variance does not apply to 2x2 contingency tables for the Fisher exact test.

Is the variance similar between the groups that are being statistically compared?	Graphs show SEM, they are broadly similar.

C- Reagents

6. To show that antibodies were profiled for use in the system under study (assay and species), provide a citation, catalog number and/or clone number, supplementary information or reference to an antibody validation profile. e.g., Antibodypedia (see link list at top right), 1DegreeBio (see link list at top right).	The rabbit anti-SARS-CoV-2 nucleocapsid antibody used in this study for viral focus assays was obtained from Sinobiological, distributed by Thermo Fisher, Cat #40143-R001). The use of this antibody for this assay is first described in the following publication: Graham et al, 202 (PMID: 33072323).
7. Identify the source of cell lines and report if they were recently authenticated (e.g., by STR profiling) and tested for mycoplasma contamination.	Vero E6 and Vero E6-TMPRSS2 cells were routinely tested for mycoplasma contamination by DAPI staining, no cytoplasmic staining was observed. Vero E6 cells were obtained directly from Dr. J.L. Whitton, Vero E6 TMPRSS-2 cells were obtained from the JCRB Cell Bank (JCRB No. JCRB1819). Neither cell line was recently authenticated.

* for all hyperlinks, please see the table at the top right of the document

D- Animal Models

8. Report species, strain, gender, age of animals and genetic modification status where applicable. Please detail housing and husbandry conditions and the source of animals.	NA
9. For experiments involving live vertebrates, include a statement of compliance with ethical regulations and identify the committee(s) approving the experiments.	NA
10. We recommend consulting the ARRIVE guidelines (see link list at top right) (PLoS Biol. 8(6), e1000412, 2010) to ensure that other relevant aspects of animal studies are adequately reported. See author guidelines, under 'Reporting Guidelines'. See also: NIH (see link list at top right) and MRC (see link list at top right) recommendations. Please confirm compliance.	NA

E- Human Subjects

11. Identify the committee(s) approving the study protocol.	The use of deindentified positive specimens for the above study was approved by the University of Washington Institutional Review Board under a consent waiver (STUDY00010205)
12. Include a statement confirming that informed consent was obtained from all subjects and that the experiments conformed to the principles set out in the WMA Declaration of Helsinki and the Department of Health and Human Services Belmont Report.	The use of deindentified samples in this study was approved by the University of Washington Institutional Review Board under a consent waiver, and were determined to be exempt.
13. For publication of patient photos, include a statement confirming that consent to publish was obtained.	There are no photos in this study.
14. Report any restrictions on the availability (and/or on the use) of human data or samples.	The data in this paper is available in the supplemental dataset. There is no remaining volume of the clinical specimans or extracted RNA used in this work.
15. Report the clinical trial registration number (at ClinicalTrials.gov or equivalent), where applicable.	NA
16. For phase II and III randomized controlled trials, please refer to the CONSORT flow diagram (see link list at top right) and submit the CONSORT checklist (see link list at top right) with your submission. See author guidelines, under 'Reporting Guidelines'. Please confirm you have submitted this list.	NA
17. For tumor marker prognostic studies, we recommend that you follow the REMARK reporting guidelines (see link list at top right). See author guidelines, under 'Reporting Guidelines'. Please confirm you have followed these guidelines.	NA

F- Data Accessibility

18: Provide a "Data Availability" section at the end of the Materials & Methods, listing the accession codes for data generated in this study and deposited in a public database (e.g. RNA-Seq data: Gene Expression Omnibus GSE39462, Proteomics data: PRIDE PXD000208 etc.) Please refer to our author guidelines for 'Data Deposition'.<br><br>Data deposition in a public repository is mandatory for:<br>a. Protein, DNA and RNA sequences<br>b. Macromolecular structures<br>c. Crystallographic data for small molecules<br>d. Functional genomics data<br>e. Proteomics and molecular interactions	All the data is included in the manuscript and supplemental materials.
19. Deposition is strongly recommended for any datasets that are central and integral to the study; please consider the journal's data policy. If no structured public repository exists for a given data type, we encourage the provision of datasets in the manuscript as a Supplementary Document (see author guidelines under 'Expanded View' or in unstructured repositories such as Dryad (see link list at top right) or Figshare (see link list at top right).	Data is available in the supplemental dataset.
20. Access to human clinical and genomic datasets should be provided with as few restrictions as possible while respecting ethical obligations to the patients and relevant medical and legal issues. If practically possible and compatible with the individual consent agreement used in the study, such data should be deposited in one of the major public access-controlled repositories such as dbGAP (see link list at top right) or EGA (see link list at top right).	Not applicable for this data type.
21. Computational models that are central and integral to a study should be shared without restrictions and provided in a machine-readable form. The relevant accession numbers or links should be provided. When possible, standardized format (SBML, CellML) should be used instead of scripts (e.g. MATLAB). Authors are strongly encouraged to follow the MIRIAM guidelines (see link list at top right) and deposit their model in a public database such as Biomodels (see link list at top right) or JWS Online (see link list at top right). If computer source code is provided with the paper, it should be deposited in a public repository or included in supplementary information.	The source code used to generate the ROC curves is available at github.com/emilybrucelab.

G- Dual use research of concern

22. Could your study fall under dual use research restrictions? Please check biosecurity documents (see link list at top right) and list of select agents and toxins (APHIS/CDC) (see link list at top right). According to our biosecurity guidelines, provide a statement only if it could.	No
